# 120 A Review of Certified Burn Therapist’s Involvement in JBCR

**DOI:** 10.1093/jbcr/irad045.093

**Published:** 2023-05-15

**Authors:** Renee Warthman, Derek Murray, Audrey O'Neil

**Affiliations:** Arizona Burn Center at Valleywise Health, Phoenix, Arizona; Arizona Burn Center Valleywise Health, Phoenix, Arizona; Richard M. Fairbanks Burn Center, Indianapolis, Indiana; Arizona Burn Center at Valleywise Health, Phoenix, Arizona; Arizona Burn Center Valleywise Health, Phoenix, Arizona; Richard M. Fairbanks Burn Center, Indianapolis, Indiana; Arizona Burn Center at Valleywise Health, Phoenix, Arizona; Arizona Burn Center Valleywise Health, Phoenix, Arizona; Richard M. Fairbanks Burn Center, Indianapolis, Indiana

## Abstract

**Introduction:**

Burn therapist certification (BT-C) was introduced in 2018 to recognize therapists with specialized skill in burn rehabilitation and promote quality burn therapy. There are currently 32 BT-Cs, representing a small fraction of therapists who rehabilitate burn patients. Barriers to certification described in a Letter to the Editor by O’Neil in 2022 included an indifference to therapy research and program development. Despite efforts to call for additional support of therapy advancement and research, therapist publications continue to be sparse. This review analyzed BT-C therapists involved in publications as one indicator of a positive impact on the field.

**Methods:**

A review of therapist-authored manuscripts and abstracts published in the Journal of Burn Care and Research from 2018-2022 was performed. Therapy authored publications included any manuscript or abstract with a therapist listed as a lead or contributing author. Publications with only SLP, PTA, or OTA credentials were excluded. Therapists were only considered to be certified if they had achieved BT-C at the time of publication.

**Results:**

BT-C make up not more than 15% of therapists practicing in burns, yet the percentage of therapy-authored manuscripts published from 2018-2022 with a BT-C credited author is 50%. When examining supplemental editions, 31% of accepted therapy-authored submissions from 2018-2022 included a BT-C therapist. The table compares the percentage of therapy-authored manuscripts and therapy-authored submissions from BT-C versus the percentage of BT-C using the 215 identified PT and OT members from 2008 as a conservative estimate of the floor of the number of PT and OT providing routine burn care, with the current count of BT-C at 32.

**Conclusions:**

Estimations of the number of PT and OT working in burn rehabilitation are difficult to determine. It is assured that therapists providing care to burn patients routinely are larger in number than those who are active members in the American Burn Association in any given year. One publication revealed 215 active PT and OT members in 2008. Despite BT-C therapists making up a small percentage of the therapy community working in burn rehabilitation, BT-C therapists contributed to half of therapy publications and almost a third of abstract submissions in each area respectively, indicating that certified burn therapists have a profound, and disproportionately large, impact on formal investigation and research.

**Applicability of Research to Practice:**

Currently there is a lack of objective information that can be presented to administrators to validate support of the burn therapist certification. Supporting certification may promote quality care through involvement in research furthering best practice.

